# Effectiveness of a Blended Intervention to Promote Physical Activity Among Office Employees: Randomized Controlled Trial

**DOI:** 10.2196/80249

**Published:** 2026-05-22

**Authors:** Yan Sun, Yang Gao, Alison Y T Ou, Xueying Wang, Yaojie Xie, Xiang-yan Chen, Shirley S M Fong, Wendy Yajun Huang, Chun-Qing Zhang, Julien S Baker, Martin S Hagger

**Affiliations:** 1 Department of Sports and Health Sciences, Academy of Wellness and Human Development, Hong Kong Baptist University, Academic and Administration Building, Baptist University Road Campus, Hong Kong, China (Hong Kong), 852 3411 7770; 2 School of Nursing, The Hong Kong Polytechnic University, Hong Kong, China (Hong Kong); 3 Division of Science, Engineering, and Health Studies, College of Professional and Continuing Education, The Hong Kong Polytechnic University, Hong Kong, China (Hong Kong); 4 Department of Health and Physical Education, The Education University of Hong Kong, Hong Kong, China (Hong Kong); 5 Department of Psychology, Sun Yat-sen University, Guang Zhou, China; 6 Department of Psychological Science, University of California, Merced, CA, United States; 7 Health Sciences Research Institute, University of California, Merced, CA, United States; 8 School of Applied Psychology, Griffith University, Brisbane, Australia; 9 Faculty of Sport and Health Sciences, University of Jyväskylä, Jyväskylä, Finland

**Keywords:** physical inactivity, moderate-to-vigorous physical activity, RCT, randomized controlled trial, health promotion

## Abstract

**Background:**

Regular moderate-to-vigorous physical activity (MVPA) reduces the risk of noncommunicable diseases, yet one-third of adults globally fail to meet MVPA recommendations. Office employees, among the least active groups, face heightened risks due to prolonged sedentary behavior and barriers like lack of time, fatigue, and low motivation. Although scalable, web-based interventions frequently face challenges, such as low engagement, high attrition, and limited personalization. Blended interventions, combining digital tools with interactive components, show promise but lack robust evidence among office employees.

**Objective:**

This study aimed to evaluate the effectiveness of a theory- and evidence-based blended intervention in increasing MVPA among office employees.

**Methods:**

This 24-week, 3-arm randomized controlled trial included 141 office employees, randomized equally into blended (web-based+electronic workshops [e-workshops]), web-based only, and control groups. The intervention was developed using the intervention mapping framework, ensuring a systematic, evidence- and theory-based design tailored to the needs of the target population. All participants accessed a study website; the control group used the “Library” module with general MVPA information, while intervention groups also accessed a tailored, theory-driven “Intervention” module. The blended group additionally attended 3 live e-workshops (weeks 2, 4, and 8). MVPA was objectively measured using hip-worn accelerometers at baseline (T1), postintervention (12 wk, T2), and follow-up (24 wk, T3). Retention, engagement, and acceptability were assessed. Analyses followed the intention-to-treat principle, and generalized linear mixed models were used to evaluate between-group differences at T2 and T3, adjusted for baseline MVPA, sex, and ActiGraph LEAP (Ametris) wear time.

**Results:**

A total of 141 participants (mean age 32.1, SD 9.2 y; 75/141, 53.2% female) were randomized evenly across groups with comparable baseline characteristics, except for sex (*P=.*002). The blended group showed significantly greater MVPA increases than the control at T2 (*β* coefficient=0.247, 95% CI 0.013‐0.480; *P*=.04) and T3 (*β* coefficient=0.373, 95% CI 0.139‐0.607; *P=.*002), and greater increases than the web-based at T2 (*β* coefficient=0.287, 95% CI 0.062‐0.512; *P=.*01) and T3 (*β* coefficient=0.368, 95% CI 0.138‐0.597; *P=.*002). Sensitivity analyses confirmed the robustness of these findings. Retention and engagement rates were 83% and 56%, respectively. Participants reported high acceptability across all domains.

**Conclusions:**

A blended intervention integrating tailored web content with interactive e-workshops significantly increased MVPA at T2 and T3 compared with both web-based and control groups. This success stems from the rigorous intervention mapping framework, which systematically identified effective behavior change and habit formation theories, while the blended approach addressed key limitations of traditional and web-based methods. These findings underscore the potential of evidence- and theory-based blended approaches to address common barriers to MVPA and promote sustainable MVPA in workplace settings, offering promising opportunities for broader scalability and personalization.

## Introduction

Noncommunicable diseases (NCDs) pose a significant global health crisis, accounting for 41 million deaths annually, or 74% of all deaths worldwide [1]. Cardiovascular diseases, cancers, chronic respiratory diseases, and diabetes are the leading contributors, driven by behavioral risk factors such as smoking, physical inactivity, unhealthy diets, and excessive alcohol consumption [2]. Among these, physical inactivity is a major contributor to NCDs and premature mortality. Regular engagement in moderate-to-vigorous physical activity (MVPA) significantly reduces the risk of cardiovascular diseases, cancers, diabetes, obesity, and mental health disorders [3]. However, nearly one-third of adults globally (1.8 billion) fail to meet the World Health Organization’s (WHO) MVPA recommendations, making insufficient MVPA the fourth leading risk factor for global mortality [2].

This issue is particularly pronounced among office employees, who spend over two-thirds of their workday in sedentary activities and often remain inactive during leisure time [4-6]. Consequently, office employees are among the least active occupational groups, facing heightened risks of NCDs and premature mortality [4]. In Hong Kong, only 46.2% (2944/6368) of adults (age: 17-79) meet global MVPA recommendations—significantly below the global average of 75% [7]. Common barriers, including lack of time, fatigue, and low motivation, further prevent office employees from incorporating MVPA into their routines [8].

The COVID-19 pandemic exacerbated physical inactivity globally. Movement restrictions, lockdowns, and the transition to remote work dramatically reduced opportunities for MVPA [9,10]. In Hong Kong, 70.4% (444/631) of adults did not meet MVPA guidelines during the pandemic [11]. Office employees, in particular, saw activity levels decline further, as remote work reduced MVPA levels in domains such as leisure time and transportation [12-14].

Web-based interventions have gained attention for their scalability and cost-effectiveness [15], with evidence showing positive effects on MVPA levels among working adults. However, their long-term effectiveness remains inconclusive due to small effect sizes, challenges such as low engagement, high attrition, and a lack of interactive or personalized components [16]. These challenges suggest that web-based interventions alone are insufficient for long-term behavior change, particularly in real-world settings. Interactive elements, such as group sessions and one-on-one interviews, have been shown to enhance engagement and effectiveness, but their integration into workplace interventions remains limited [17].

Blended interventions, which integrate digital tools (eg, web-based self-monitoring and feedback systems) with interactive elements, present a promising alternative by providing personalized support, real-time feedback, and social interaction [17]. Nevertheless, systematic reviews, such as Hohberg et al [17], highlight significant methodological heterogeneity in blended interventions, which can be attributed to variations in duration, intensity, theoretical foundations, and component integration. Such inconsistencies limit definitive conclusions about their efficacy, especially for specific populations, such as office employees. Therefore, there is an urgent call for systematically designed interventions that address these limitations by using robust, theory-based, and evidence-based frameworks.

To address these gaps, this study evaluates the effectiveness of a novel blended intervention designed to increase MVPA, improve health outcomes, and enhance work productivity among office employees. The intervention was systematically developed using the intervention mapping (IM) framework, which integrates evidence-based practices and behavior change theories to guide the design of interventions that are specifically tailored to the needs and barriers of the target population [18]. By combining web-based self-monitoring tools with interactive electronic workshops (e-workshops), the intervention aims to overcome key limitations of both web-based and traditional approaches. Importantly, the use of the IM framework ensures a structured and comprehensive approach to intervention design, addressing the methodological heterogeneity observed in previous studies. The specific objectives of this study are (1) to assess changes in actigraphy-measured MVPA levels among participants, and (2) to examine differences in engagement and retention rates across intervention groups.

## Methods

### Study Design

This study adhered to the CONSORT (Consolidated Standards of Reporting Trials) 2010 guidelines for randomized controlled trials (RCT) [19,20]. The original study was designed as a cluster-RCT [21]. However, disruptions caused by the COVID-19 pandemic and recruitment challenges necessitated its adaptation to a standard RCT with convenience sampling. Key changes included adjusting the estimated sample size from 495 (cluster-RCT design) to 135 (RCT design), replacing face-to-face workshops with e-workshops conducted via Zoom (Zoom Communications), and omitting workplace-level environmental changes.

**Figure 1.**
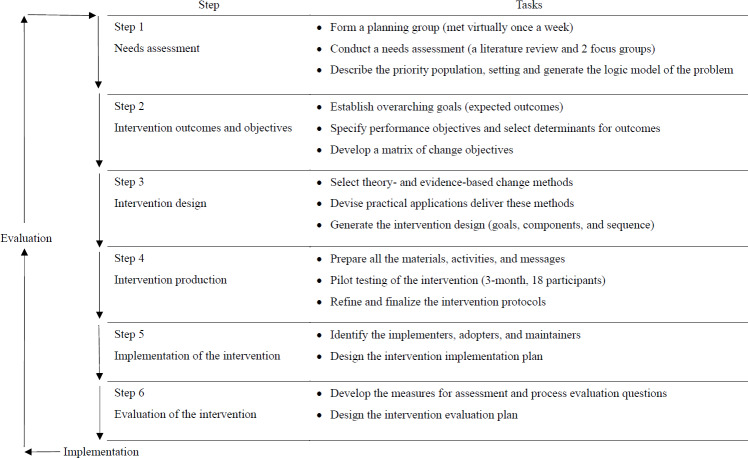
Overview of intervention mapping steps and corresponding tasks.

The intervention was developed using the IM framework, a systematic and iterative approach for designing evidence-based health promotion programs. The IM framework comprises 6 steps, with the first 4 steps focusing on the development of the intervention. These steps provided a structured, theory-based, and evidence-based process to create a tailored intervention addressing the needs of the target population. Figure 1 summarizes the IM framework and the tasks involved in each step.

Step 1 identified the health problem, its determinants, and the needs of the target population. Step 2 defined the intervention’s expected outcomes, specified performance objectives, and developed a matrix of change objectives. In step 3, evidence-based behavior change methods were selected and translated into practical applications to address these objectives. Finally, step 4 focused on preparing and pilot-testing intervention materials to ensure feasibility, acceptability, and usability. This process enabled the development of a comprehensive and adaptable intervention, even under the constraints posed by the COVID-19 pandemic.

This study was a 24-week, 3-arm RCT. Participants were randomly allocated to one of the 3 groups—a web-based intervention group, a blended intervention group (combining web-based with e-workshops), or a control group. Assessments were conducted at baseline (T1), postintervention (T2, 12 wk), and follow-up (T3, 24 wk). All participants accessed the same website, which consisted of 2 main modules, a “Library” module with generic MVPA information and an “Intervention” module with personalized, theory-driven content (accessible only to the web-based and blended intervention groups). Additionally, participants in the blended intervention group attended 3 e-workshops at weeks 2, 4, and 8. All participants received fortnightly reminders to engage with the website.

### Ethical Considerations

This study was approved by the Research Ethics Committee of Hong Kong Baptist University (approval HASC/17-18/0529). All participants provided electronic informed consent via the QuestionPro platform after being fully informed of the study’s objectives, procedures, potential risks, and benefits. The informed consent process also granted permission for secondary analysis of the collected data without requiring additional consent.

Identifiable personal data will be destroyed after the study is completed, while deidentified datasets will be retained for research purposes and can be requested from the corresponding author (YG) upon reasonable request. Participants were compensated with HK $50 (approximately US $6.40) coupons for each completed evaluation session. Additionally, those who completed all study requirements were entered into a lottery to win a HK $1000 (approximately US $128; quota: 5) coupon. As part of the study benefits, participants also received a personalized physical activity and health tracking report summarizing their progress and providing actionable insights. No identifiable images or information about participants are included in the manuscript or supplementary materials.

### Eligibility Criteria and Recruitment Process

Eligible participants were office employees in Hong Kong aged 18 years or older, engaging in less than 150 minutes of MVPA per week according to WHO guidelines, assessed using the short-form International Physical Activity Questionnaire [22]. To ensure safety, participants completed the Physical Activity Readiness Questionnaire, and those who answered “yes” to any question were required to obtain medical clearance before participation. Individuals enrolled in other MVPA promotion programs or with conditions preventing physical activity were excluded.

Recruitment was conducted remotely during the pandemic of COVID-19 (September-December 2022) when many employees were working from home. A prerandomized list of 440 companies, representing 20% of eligible Hong Kong companies with more than 100 employees, was obtained from the Hong Kong Census and Statistics Department. Due to pandemic-related concerns, most companies declined participation or did not respond, with only 4 agreeing to disseminate study materials to their office employees. Social media platforms, such as Facebook (Meta), Instagram (Meta), and WeChat (Tencent Holdings Limited), were also used, resulting in the recruitment of 88 participants. Referrals were encouraged through unique tracking links shared by interested individuals with colleagues, friends, or family members who met the eligibility criteria, which led to the recruitment of an additional 27 participants. In total, 172 individuals were assessed for eligibility, and 141 participants (82%) completed baseline enrollment.

### Randomization and Allocation

Participants were randomly assigned to the 3 study groups using block randomization with a fixed block size of 6 to ensure balanced allocation throughout enrollment. The randomization sequence was generated using a computer-based random number generator and administered by an independent research team member who was not involved in the intervention or outcome assessment. Allocation concealment was ensured, and outcome assessors remained blinded to group assignments.

### Sample Size Estimation

The sample size was calculated using repeated measures ANOVA within-between interaction approach (G*Power, version 3.1.9.6), where a power set at 0.80 and an α at .05, with 3 groups and 3 measurement waves. An effect size of 0.30 (Cohen *d*) was chosen based on a similar study in terms of design and intervention components [23], and on a median effect size of 0.30 (IQR 0.16-0.38), calculated from 19 studies reporting Cohen *d* in a recent systematic review of blended interventions [17]. To account for an expected attrition rate of 30%, the required sample size was estimated at 135 participants, with 45 participants allocated to each group [24].

### Intervention

#### Control Group

Participants in the control group accessed only the “Library” module of the website, which provided general, nontailored information on MVPA, health, and work productivity. This content consisted of 18 short essays, delivered every 2 weeks across 6 sessions, covering topics such as recommended MVPA levels, the benefits of MVPA, and the risks of inactivity.

In total, 3 behavior change techniques (BCTs) were embedded in the materials, focusing on the consequences of health, social, and emotional outcomes (codes 5.1, 5.3, and 5.6, respectively) [25]. For instance, 1 essay (#3 in session 1) addressing the consequences of health outcomes (code 5.1) explained the risks of inactivity (eg, heart disease, diabetes, and cancer), and outlined benefits such as improved cardiovascular health and better sleep, supported by evidence-based data. Another essay (#4 in session 2) focused on the social benefits of physical activity (code 5.3), such as meeting new people, fostering teamwork, and building support systems through group activities like sports or fitness classes. It described how exercising with others can create a sense of belonging and improve teamwork skills. Additionally, an essay (#5 in session 2) explored emotional benefits (code 5.6), explaining how physical activity can boost mood and reduce stress, depression, and anxiety by releasing endorphins and serotonin, with examples like running, yoga, or brisk walking.

After the study concluded, control group participants were granted access to the “Intervention” section for exposure to the web-based intervention content.

#### Web-Based Intervention Group

In addition to accessing the “Library,” participants in the web-based intervention group engaged with the “Intervention” module of the website, following the same schedule as the control group. Within this module, participants answered questions and received individually tailored feedback on their MVPA levels, behavioral determinants, and social and environmental factors. The feedback was based on a theoretical framework that integrates motivational, volitional, and habit-formation processes, drawing from dual-process theories and habit formation theories to support both behavior change and maintenance [26-28].

A total of 31 BCTs were selected and integrated based on these 3 processes [25,29]. For example, in session 3**,** participants were guided to stay motivated by identifying their main barriers to MVPA and developing tailored coping strategies. Suggestions included trying lighter activities, such as yoga or walking, to address fatigue, or scheduling short, manageable bouts of activity (eg, 10-min brisk walks) for busy days. Participants were also reminded of the mental and emotional benefits of exercise, such as improved mood and reduced stress, to reinforce motivation. Further session-specific details, including questions, feedback, and BCTs, are provided in Table S1 in Multimedia Appendix 1.

#### Blended Intervention Group

In addition to accessing 2 modules on the website, participants in the blended intervention group attended 3 live e-workshops via Zoom, scheduled for weeks 2, 4, and 8. Each e-workshop lasted 30 to 45 minutes and involved a facilitator, a moderator, and 4 to 10 participants. These workshops were designed to provide direct and immediate individualized feedback, facilitate the sharing of experiences among participants, and encourage adherence to the intervention [15].

To enhance the effectiveness of the intervention, 4 additional BCTs were integrated into the workshops: problem solving (code 1.2), instruction on how to perform the behavior (code 4.1), reattribution (code 4.3), and mental rehearsal of successful performance (code 15.2) [25]. For example, during the third workshop, participants applied problem solving (code 1.2) by identifying barriers to MVPA (eg, bad weather or lack of time) and collaboratively developing coping strategies using the “If…then…” framework (eg, “If the weather is bad, then I will do a 20-min yoga session at home”). The facilitator provided real-time feedback to ensure practicality, while the group setting encouraged idea sharing and accountability, enhancing engagement compared with online modules.

### Outcome Measures

MVPA was objectively measured by a hip-worn accelerometer (ActiGraph, Models wGT3X-BT) at T1-T3, and data were analyzed by using ActiLife software v6.13.4 (ActiGraph LLC). The participants were instructed to wear an accelerometer on the right hip during waking hours for 7 consecutive days, except when bathing, performing water-based activities, and sleeping [30]. Freedson cutoff points were used to classify VPA, MPA, and light-intensity physical activity [31]. Data of at least 10 hours per day for a minimum of 3 days (including at least 1 weekend day) were regarded as valid and included in data analysis [32,33]. Then, the total weekly MVPA was calculated by multiplying the average daily MVPA levels by 7.

The retention rate (%) was calculated as participants at the end of the intervention divided by the participants at baseline.

The engagement rate (%) was calculated by the number of intervention sessions attended by participants divided by the total number of sessions provided to them. In the blended group, engagement rates were calculated for web-based sessions and e-workshop sessions separately and in combination.

The acceptability of the intervention was evaluated using a 5-point Likert scale (from “1=strongly dissatisfied” to “5=strongly satisfied”) across several dimensions, such as ease of use, understandability, usefulness, enjoyability, time acceptability, and overall satisfaction [34,35]. Statements included, for example, “The content of the intervention is understandable” and “The time taken to complete the content of the intervention was acceptable.” For the evaluation of the e-workshops, similar statements were adapted. Responses were grouped, combining “agree” and “strongly agree” into one percentage, and “disagree” and “strongly disagree” into another, to reflect participant agreement or disagreement.

### Data Analysis

MVPA (min/wk) was summarized as mean and SD at each assessment time point based on valid ActiGraph data. Primary intervention effects at T2 and T3 were evaluated under an intention-to-treat (ITT) framework using generalized linear mixed models with a γ distribution and a log link. Participants were analyzed as randomized. The generalized linear mixed models included fixed effects for group, time, and the group×time interaction, with participant treated as a random effect. Models were adjusted for sex, ActiGraph wear time, and baseline MVPA. Between-group intervention effects were estimated as differences in change from baseline using the group×time interaction (difference-in-differences); pairwise contrasts were obtained by changing reference groups where needed. For completeness, estimated within-group changes from baseline are also presented.

At baseline, MVPA values were unavailable for 15 participants because ActiGraph recordings did not meet the prespecified wear-time validity criteria. These baseline values were replaced using within-group mean imputation for the primary analyses [36]. Sensitivity analyses used multiple imputation (MI) for MVPA across all 3 time points, generating 5 imputations [37,38]. The MI model incorporated baseline values and other variables associated with MVPA to improve estimation accuracy.

Retention and engagement rates were compared across groups using chi-square tests. Statistical significance was set at *P*<.05, with 95% CIs reported. All analyses were performed in IBM SPSS Statistics (version 29.0.1).

## Results

**Figure 2.**
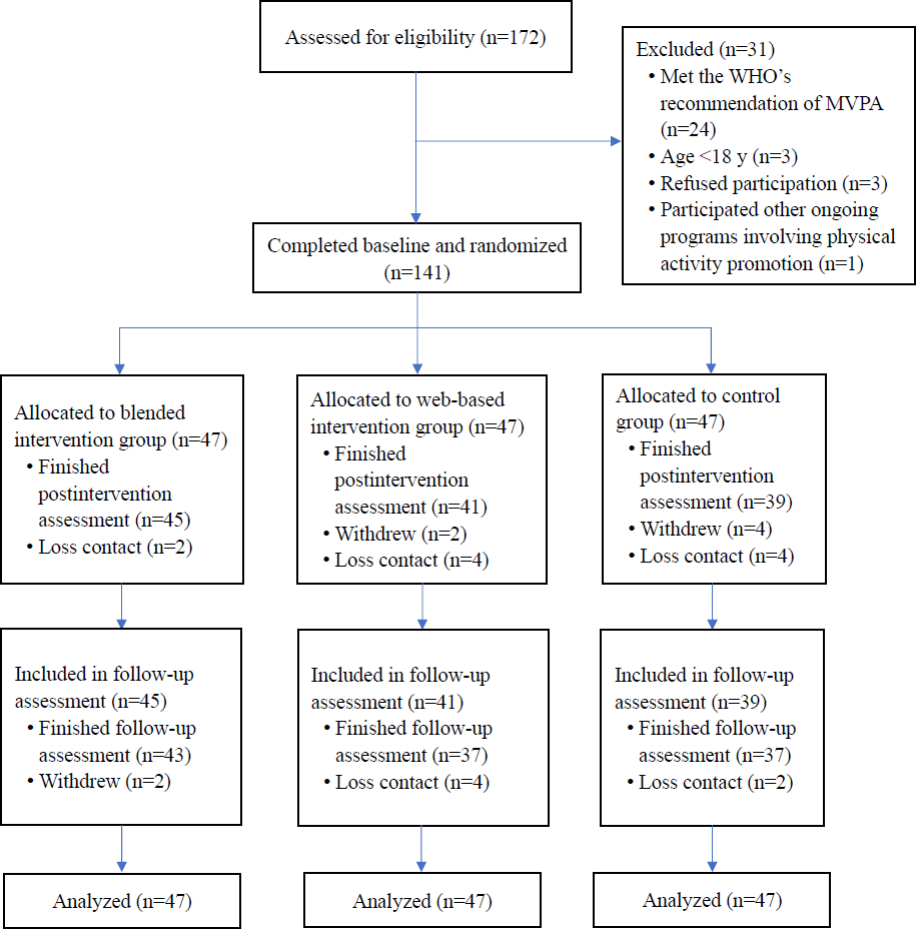
Flow diagram of the study. MVPA: moderate-to-vigorous physical activity; WHO: World Health Organization.

A total of 172 office employees were assessed for eligibility, and 141 of them completed the baseline assessment (mean age 32.05, SD 9.22 y; 75/141, 53.2% female), with each group consisting of 47 participants. The participant flow is illustrated in Figure 2. The baseline sociodemographic characteristics are presented in Table 1. There were no demographic differences between participants who completed 3 assessments and those who were lost to follow-up.

**Table 1.** Sociodemographic characteristics of the participants at baseline.

Characteristic	Total (n=141)	Blended (n=47)	Web-based (n=47)	Control (n=47)
Age (y), mean (SD)	32.05 (9.22)	33.15 (7.77)	32.14 (10.27)	30.57 (9.38)
Sex, n (%)				
Male	66 (46.8)	19 (40.4)	27 (57.4)	20 (42.6)
Female	75 (53.2)	28 (59.6)	20 (42.6)	27 (57.4)
Education level, n (%)				
Diploma or below	28 (19.9)	10 (21.3)	9 (19.1)	9 (19.1)
Bachelor or above	113 (80.1)	37 (78.7)	38 (80.9)	38 (80.9)
Job category, n (%)				
Administrators or associate professionals	84 (59.6)	28 (59.6)	33 (70.2)	23 (48.9)
Clerical or services related	57 (40.4)	19 (40.4)	14 (29.8)	24 (51.1)
Marital status, n (%)				
Single	88 (62.4)	27 (57.4)	29 (61.7)	32 (68.1)
Not single	53 (37.6)	20 (42.6)	18 (38.3)	15 (31.9)
Monthly salary (HKD), n (%)				
<20,000 (<US $2560)	52 (36.9)	13 (27.7)	16 (34)	23 (48.9)
≥20,000 (≥US $2560)	89 (63.1)	34 (72.3)	31 (66)	24 (51.1)

Table 2 presents means and SDs for actigraphy-measured MVPA (min/wk) and ActiGraph wear time (min/d) by group at three time points. Descriptive statistics are based on valid recordings only (complete cases for each time point). Sample sizes differ across time points because some recordings did not meet the prespecified wear-time validity criteria and some assessments were missing (including dropout).

**Table 2.** Mean (SD) for actigraphy-measured MVPA^a^ (min/wk) and ActiGraph wear time (min/d) by group at 3 time points. Values are presented as mean (SD) based on valid ActiGraph data. Sample sizes (n) vary across time points due to invalid recordings and missing assessments (including dropout).

Time point	n	MVPA (min/wk), mean (SD)	Wear time (min/d), mean (SD)
Baseline			
Blended	43	163.17 (80.39)	729.95 (77.04)
Web-based	45	163.47 (76.72)	740.92 (104.53)
Control	38	159.19 (79.79)	699.03 (70.55)
Postintervention			
Blended	39	244.80 (115.37)	725.50 (71.44)
Web-based	37	185.78 (60.95)	727.84 (77.53)
Control	34	183.71 (62.22)	717.78 (104.35)
Follow-up			
Blended	40	268.00 (103.20)	754.67 (77.14)
Web-based	34	197.70 (73.24)	693.63 (60.56)
Control	33	194.56 (83.86)	697.33 (66.96)

a MVPA: moderate-to-vigorous physical activity.

Table 3 presents within-group changes and between-group differences in changes in actigraphy-measured MVPA at T2 and T3 relative to T1, using both ITT and sensitivity analyses. In the ITT analysis, MVPA increased significantly from T1 to T2 and T3 within all 3 groups (all *P*<.01). For between-group differences in change, the blended group showed significantly greater improvements than the control group at T2 (*β* coefficient=0.247, 95% CI 0.013‐0.480; *P=.*04) and than the web-based group at T2 (*β* coefficient=0.287, 95% CI 0.062‐0.512; *P=.*01) at T2. These effects were maintained at T3, with the blended group continuing to demonstrate greater improvements compared with both the control group (*β* coefficient=0.373, 95% CI 0.139‐0.607; *P=.*002) and the web-based group (*β* coefficient=0.368, 95% CI 0.138‐0.597; *P=.*002). No significant differences in changes were observed between the web-based and control groups at either time point.

Sensitivity analyses using MI displayed a similar pattern, with the blended group demonstrating greater improvements than both the control and web-based groups at both T2 and T3 (all *P<.*001). These findings are consistent with the primary ITT analysis and support the robustness of the conclusions.

**Table 3.** GLMM^a^ estimates for actigraphy-measured MVPA^b^ (within-group changes from T1 to T2 and T3 and between-group differences in change [ITT^c^ {primary} and sensitivity analyses^d^]).

Effect	Standardized β coefficient (95% CI)	SE	*t* test (*df*)	*P* value
ITT estimates				
Baseline to postintervention				
Within blended	0.500 (0.342 to 0.658)	0.080	6.228 (327)	<.001
Within web-based	0.213 (0.053 to 0.373)	0.081	2.617 (327)	.009
Within control	0.253 (0.064 to 0.442)	0.087	2.901 (327)	.006
Blended versus control	0.247 (0.013 to 0.480)	0.119	2.080 (327)	.04
Web-based versus control	−0.040 (−0.275 to 0.194)	0.119	−0.339 (327)	.74
Blended versus web-based	0.287 (0.062 to 0.512)	0.114	2.515 (327)	.01
Baseline to follow-up				
Within blended	0.633 (0.452 to 0.814)	0.080	7.868 (327)	<.001
Within web-based	0.265 (0.077 to 0.453)	0.084	3.162 (327)	.003
Within control	0.260 (0.063 to 0.456)	0.088	2.965 (327)	.006
Blended versus control	0.373 (0.139 to 0.607)	0.119	3.137 (327)	.002
Web-based versus control	0.005 (−0.233 to 0.244)	0.121	0.045 (327)	.96
Blended versus web-based	0.368 (0.138 to 0.597)	0.117	3.149 (327)	.002
Sensitivity estimates				
Baseline to postintervention				
Within blended	0.500 (0.436 to 0.563)	0.032	15.452 (2022)	<.001
Within web-based	0.213 (0.148 to 0.277)	0.033	6.494 (2022)	<.001
Within control	0.253 (0.184 to 0.322)	0.035	7.197 (2022)	<.001
Blended versus control	0.247 (0.153 to 0.340)	0.048	5.160 (2022)	<.001
Web-based versus control	−0.040 (−0.135 to 0.054)	0.048	−0.839 (2022)	.40
Blended versus web-based	0.287 (0.197 to 0.377)	0.046	6.237 (2022)	<.001
Baseline to follow-up				
Within blended	0.633 (0.560 to 0.705)	0.032	19.566 (2022)	<.001
Within web-based	0.265 (0.189 to 0.340)	0.034	7.858 (2022)	<.001
Within control	0.260 (0.181 to 0.338)	0.035	7.370 (2022)	<.001
Blended versus control	0.373 (0.279 to 0.467)	0.048	7.804 (2022)	<.001
Web-based versus control	0.005 (−0.090 to 0.101)	0.049	0.109 (2022)	.91
Blended versus web-based	0.368 (0.276 to 0.460)	0.047	7.834 (2022)	<.001

a GLMM: generalized linear mixed models with a Gamma distribution and log link. Models included fixed effects for group, time, and their interaction (group×time), adjusted for sex, wear time, and baseline moderate-to-vigorous physical activity. Between-group rows report coefficients for the group×time interaction only. Main effects (group and time), covariates (baseline MVPA, wear time, and sex), and other interaction terms were included in the models but are not displayed.

b MVPA: moderate-to-vigorous physical activity.

c ITT: intention-to-treat. Primary analyses followed the intention-to-treat principle using all available repeated measures.

d Sensitivity analyses used multiple imputation with pooled estimates across imputations.

Retention and engagement rates across groups are presented in Table 4. The overall retention and engagement rates were 83% and 56%, respectively, with no significant differences observed between groups. In the blended group, the overall engagement rate was 56%, consisting of 61% for web-based sessions and 46.1% for e-workshops.

Participants across all groups reported high levels of acceptability for the web-based sessions, with strong agreement observed for ease of use, understandability, usefulness, enjoyability, time acceptability, and satisfaction (Table S2 in Multimedia Appendix 2). Ease of use and understandability were rated highly, with over 90% agreement in all groups. Usefulness and satisfaction also showed high agreement, exceeding 88% across groups. Enjoyability had slightly lower agreement rates but remained above 77% for all groups. Time acceptability was rated positively by most participants, with agreement rates above 88% in all groups.

No significant harms or unintended effects were reported in any of the intervention groups. Participants did not report any adverse events related to the intervention, and all activities were conducted within safe and acceptable parameters.

**Table 4.** Retention and engagement rates of the intervention by group.

Outcomes	Total, n/N (%)	Blended, n/N (%)	Web-based, n/N (%)	Control, n/N (%)	Chi-square (*df*)	*P* value
Retention	117/141 (83)	43/47 (91.5)	37/47 (78.7)	37/47 (78.7)	3.6 (2)	.16
Engagement	553/987 (56)	237/423 (56)	156/282 (55.3)	160/282 (56.7)	0.1 (2)	.94

## Discussion

### Principal Findings

This study evaluated the effectiveness of a blended intervention, combining web-based content with interactive e-workshops, in promoting MVPA among physically inactive office employees. The findings demonstrated that the blended intervention significantly increased MVPA levels at both T2 and T3, outperforming the web-based intervention and the control group. Retention rates were high across all groups, with the blended group achieving the highest rate, further supporting the feasibility and acceptability of the intervention. These results underscore the potential of blended approaches in addressing physical inactivity among office employees, a population particularly vulnerable to sedentary behaviors.

The significant improvements in MVPA levels observed in the blended group at both T2 and T3 underscore the effectiveness of integrating web-based tools with interactive e-workshops. Compared with the web-based and control groups, the blended intervention achieved the most substantial increases in MVPA, highlighting its potential to surpass web-only or traditional approaches. In contrast, the web-based group showed modest improvements, while the control group exhibited minimal changes, with both groups showing significant changes at T3.

The success of the blended intervention can be attributed to its comprehensive, theory-based design, which incorporated a total of 34 BCTs targeting motivation, self-regulation, and habit formation. Previous meta-analyses have demonstrated that interventions using multiple BCTs grounded in behavioral theories tend to produce stronger effects on physical activity [39,40]. While the web-based intervention also used 31 BCTs, the blended intervention expanded upon these by incorporating 3 additional BCTs unique to the e-workshops. These additional BCTs further enhanced the intervention’s overall effectiveness by promoting interpersonal interaction, fostering peer support, and providing real-time feedback and accountability.

The group-based nature, combined with the digital person-to-person component of the e-workshops, played a pivotal role in the success of the blended intervention. The group-based format fostered interpersonal interaction and collaboration, encouraging participants to share experiences, brainstorm solutions to common barriers, and build self-efficacy [41]. Peer support within the group likely strengthened participants’ motivation and commitment, as they could learn from others’ strategies and successes, while also feeling a sense of collective accountability. At the same time, the digital person-to-person component added a human support that is often missing in purely digital interventions [42]. Unlike the impersonal nature of human-computer interactions, face-to-face communication offered warmth, empathy, and dynamic support. Facilitators were able to provide real-time feedback and adaptive guidance, promptly addressing participants’ individual challenges. Together, these features fostered a supportive and interactive environment that not only promoted encouragement, belonging, and accountability but also strengthened the application of behavior change theories targeting motivation, volition, and habit formation. By reinforcing these theoretical foundations, the blended approach amplified and consolidated the impact of the web-based content, ensuring a more robust and sustainable behavior change process.

In contrast, the web-based and control groups lacked these collaborative and interactive elements, which may explain the superior effectiveness of the blended intervention. While the web-based intervention provided valuable tools for self-regulation and goal-setting, it did not offer the same level of social reinforcement, dynamic problem-solving, or accountability as the blended approach. These findings align with previous research emphasizing the superiority of blended interventions, which effectively combine the flexibility of digital tools with the social and interactive benefits of face-to-face components [17].

The long-term improvements in MVPA levels observed at T3 suggest that the blended intervention may have facilitated long-term behavior change. This aligns with habit formation theory, which posits that consistent behaviors are more likely to be maintained when reinforced in stable contexts and supported by environmental cues [43]. Periodic e-workshops likely served as motivational “boosters,” helping participants overcome lapses and maintain engagement over time. This is particularly critical for workplace interventions, where competing demands and time constraints often challenge long-term adherence.

However, the web-based intervention alone demonstrated limited impact on MVPA levels compared with the control group, despite offering tailored feedback based on behavioral determinants. This is consistent with previous studies reporting mixed results for digital-only interventions, especially those lacking interactive or social components [36,44]. The absence of interpersonal interaction and real-time feedback in the web-based group may have reduced participants’ sense of accountability and support—both key drivers of long-term behavior change [16]. Without the reinforcement provided by workshops, participants may have struggled to translate digital content into consistent physical activity behaviors. These findings highlight the critical role of interactive components, such as group-based e-workshops, in enhancing the effectiveness of digital interventions.

The findings highlight the feasibility and acceptability of the intervention, as evidenced by high retention rates and positive participant feedback. Notably, the retention rates in this study were relatively high compared with similar interventions, which typically report rates ranging from 59% to 82% [36,45-47]. This can be attributed to the systematic and evidence-based design of the intervention using IM, which ensured the intervention was theory-driven and tailored to meet the needs of the target population [48]. Furthermore, participant retention was supported by strategies such as providing MVPA and health progress reports, as well as incentives like supermarket coupons and a grand prize for completing all outcome assessments. These measures created a structured and supportive environment, encouraging long-term participation.

Directly comparing engagement across studies is challenging due to differences in how engagement is defined and measured. While our study calculated engagement as a rate, other studies have used metrics like mean session completions or participant distributions, complicating direct comparisons. Despite this, previous studies have shown that blended interventions often achieve better engagement than platform-only or control groups. For example, 1 study found that adding coaching significantly improved session completion rates [49], while another highlighted the value of interactive components in enhancing adherence [50]. In our study, the lack of significant differences in engagement may be explained by limited participation in e-workshops, likely due to scheduling challenges and competing responsibilities during personal time, which diminished the potential advantages of the blended format. Future research could explore strategies such as offering more flexible time slots to overcome these barriers and improve engagement in blended interventions.

### Strengths and Limitations

The strengths of this study lie in its rigorous design, guided by the IM framework, which ensured a systematic and theory-driven approach to intervention development and evaluation. The use of objective measures, such as actigraphy, provided robust and reliable data on MVPA outcomes. Additionally, the high retention and engagement rates observed in the blended group demonstrate the intervention’s feasibility and acceptability in a workplace setting.

However, several limitations should be acknowledged. First, the ActiGraph device used to measure MVPA did not capture water-based activities, potentially underestimating MVPA levels among participants who engaged in swimming or similar activities. Second, the sample was skewed toward younger, highly educated, and digitally literate individuals, limiting the generalizability of the findings to other populations. Third, baseline MVPA levels in this study were relatively high, possibly due to discrepancies between self-reported activity during recruitment and objective measurements at baseline. This might have resulted in a ceiling effect, underestimating the intervention’s true potential. Finally, the study was conducted exclusively among office employees in Hong Kong, a dense urban setting with unique cultural and environmental factors, which may limit the applicability of the findings to other contexts.

### Conclusions

This study provides strong evidence for the effectiveness of a blended intervention in promoting MVPA among physically inactive office employees. By integrating web-based platforms with interactive e-workshops, the intervention effectively addressed key barriers to MVPA and achieved significant improvements in engagement, retention, and behavioral outcomes. These findings highlight the potential of blended approaches to address physical inactivity in workplace settings and beyond.

Future research should build on these findings by exploring the long-term sustainability of behavioral changes achieved through blended interventions and their scalability across diverse populations and settings. Additionally, leveraging emerging technologies, such as artificial intelligence, to deliver personalized feedback and adaptive interventions could further enhance engagement and effectiveness. Addressing these questions will be critical for advancing workplace health promotion and tackling the global physical inactivity crisis.

## Supplementary material

10.2196/80249 Multimedia Appendix 1 Timeline, questions, personally-tailored feedback, and behavior change techniques involved in 6 web-based sessions in 2 intervention groups.

10.2196/80249 Multimedia Appendix 2 Acceptability of the intervention between groups.

10.2196/80249 Checklist 1 CONSORT checklist: moderate-to-vigorous physical activity.

## Abbreviations

BCT: behavior change technique
CONSORT: Consolidated Standards of Reporting Trials
e-workshop: electronic workshop
IM: intervention mapping
ITT: intention-to-treat
MI: multiple imputation
MVPA: moderate-to-vigorous physical activity
NCDs: noncommunicable diseases
RCT: randomized controlled trial
WHO: World Health Organization
