# Supplementary material for: Effectiveness of a Blended Intervention to Promote Physical Activity Among Office Employees: Randomized Controlled Trial
Source: J Med Internet Res. 2026 May 22;28:e80249. doi: 10.2196/80249 (PMC13197156; doi:10.2196/80249)
Supplement: Multimedia Appendix 1 [file jmir-v28-e80249-s001.docx]

Appendix 1

Table S1 Timeline, questions, personally-tailored feedback, and BCTs involved in six web-based sessions in two intervention groups*

| Session/  *Time* | Questions asked | Personally-tailored feedback/  *Theoretical strategy involved* | BCTs involved/  Name *(code)*** |
| --- | --- | --- | --- |
| Session 1  *(Week 1)* | - MVPA in the past week - Goal setting - MVPA self-efficacy - Preferred MVPA routine - Habit strength of MVPA | - MVPA guideline related to the main motivation selected *(motivational strategy)* - Feedback on current MVPA level related to the main motivation *(self-regulation strategy)* - The benefits of MVPA for physical and mental health *(motivational strategy)* - Strategies to increase MVPA to meet the WHO’s recommendations based on self-efficacy level *(self-regulation strategy)* - Developing habits based on preferred activity in routine life *(habit development strategy)* - Recognising prompts related to habit strength level *(habit development strategy)* | - Goal setting (behaviour)*(1.1)* - Feedback on behaviour *(2.2)* - Information about health consequences *(5.1)* - Information about emotional consequences *(5.6)* - Behaviour substitution *(8.2)* - Prompts/cues *(7.1)* - Habit formation *(8.3)* - Verbal persuasion about   Capability *(15.1)*   - Instruction on how to perform the behaviour *(4.1)* |
| Session 2  *(Week 3)* | - MVPA in the past week - Weight - MVPA goals (both long- and short-term, open questions) - Habits and prompts - Action planning phrased with/without reference to habit (open answers) | - MVPA guidance refresher based on the main goal *(self-regulation strategy)* - MVPA progress feedback *(self-regulation strategy)* - Long- and short- term SMART goals *(motivational strategy)* - Feedback on developing habits and noticing prompts *(habit development strategy)* - Action plan *(self-regulation strategy)* - Action plan with reference to behaviour repetition in a stable context *(habit development strategy)* | - Goal setting (behaviour)*(1.1)* - Goal setting (outcome) *(1.3)* - Action planning *(1.4)* - Feedback on behaviour *(2.2)* - Self-monitoring of behaviour (2.3) - Prompts/cues *(7.1)* - Habit formation *(8.3)* - Graded tasks (8.7) - Behavioural practice/rehearsal *(8.1)* |
| Session 3  *(Week 5)* | - MVPA in the past week - Weight - Coping self-efficacy - Action plan completed - Main prompts - Action plan evaluated (open answers) | - MVPA progress feedback *(self-regulation strategy)* - Boosting confidence and staying motivated based on identified barriers *(motivational strategy)* - Evaluation and adjustment of action plan *(self-regulation strategy)* - Noticing prompts *(habit development strategy)* - Action plan *(self-regulation strategy)* - Action plan with reference to behaviour repetition in a stable context *(habit development strategy)* | - Action planning *(1.4)* - Review behaviour goal(s) *(1.5)* - Discrepancy between current behaviour and goal *(1.6)* - Review outcome goal(s) *(1.7)* - Monitoring of emotional consequences *(5.4)* - Anticipated regret *(5.5)* - Information about emotional consequences *(5.6)* - Graded tasks *(8.7)* - Prompts/cues *(7.1)* - Habit formation *(8.3)* |
| Session 4  *(Week 7)* | - MVPA in the past week - Weight - Positive social support - Negative social support - Influence of others | - MVPA progress feedback *(self-regulation strategy)* - Positive influence of others and dyadic plans *(motivational strategy)* - Positive influence of others and dyadic plans *(self-regulation strategy)* - Positive influence of others and dyadic routines *(habit development strategy)* - Negative influence of others and staying motivated when others are not supportive - Encouraging others to be active | - Review behaviour goal(s) *(1.5)* - Discrepancy between current behaviour and goal *(1.6)* - Review outcome goal(s) *(1.7)* - Social support (practical) *(3.2)* - Social support (emotional) *(3.3)* - Information about others’ approval *(6.3)* - Focus on past success *(15.3)* - Self-reward *(10.9)* - Restructuring the social environment *(12.2)* |
| Session 5  *(Week 9)* | - MVPA in the past week - Weight - Behavioural barriers - **Coping self-efficacy** - **Experience of habit development** - Re-evaluating intervention goals (open questions) - Habit development | - MVPA progress feedback *(self-regulation strategy)* - Maintaining positive habits – coping planning *(habit development strategy)* - Relapse prevention based on the main barrier selected with an emphasis on staying motivated *(motivational strategy)* - Relapse prevention based on the main barrier selected with an emphasis on staying motivated and self-regulating *(self-regulation strategy)* - Relapse prevention based on the main barrier selected with an emphasis on staying motivated/self-regulating and maintaining positive habits *(habit development strategy)* | - Problem solving *(1.2)* - Reduce prompts/cues *(7.3)* - Behaviour substitution *(8.2)* - Habit reversal *(8.4)* - Habit formation *(8.3)* - Reducing negative emotions *(11.2)* - Avoidance/ reducing exposure to cues for the behaviour *(12.3)* - Review outcome goal(s) *(1.7)* - Behavioural practice/rehearsal *(8.1)* |
| Session 6  *(Week 11)* | - MVPA in the past week - Weight - Habit strength | - MVPA progress feedback with graph *(self-regulation strategy)* - Weight changes throughout the intervention with graph *(self-regulation strategy)* - Tips to stay motivated *(motivational strategy),* to self-regulate MVPA *(self-regulation strategy),* and to follow newly developed routines *(habit development strategy)* | - Feedback on behaviour *(2.2)* - Feedback on outcome(s) of behaviour *(2.7)* - Information about health consequences *(5.1)* - Information about emotional consequences *(5.6)* - Information about others’ approval *(6.3)* - Behavioural practice/rehearsal *(8.1)* - Habit formation *(8.3)* |

Abbreviations: BCTs: behavioural change techniques; MVPA: moderate-to-vigorous physical activity

*: The table is modified from Table 1 in the original published research protocol (Sun et al., 2020). Bold font indicates new additions to the Table 1 of the protocol.

**: Names and codes of BCTs were drawn from (Michie et al., 2015).
