# Supplementary material for: Effectiveness of a Blended Intervention to Promote Physical Activity Among Office Employees: Randomized Controlled Trial
Source: J Med Internet Res. 2026 May 22;28:e80249. doi: 10.2196/80249 (PMC13197156; doi:10.2196/80249)
Supplement: Multimedia Appendix 2 [file jmir-v28-e80249-s002.docx]

Appendix 2

Table S2 Acceptability of the intervention between groups

|  | Blended | Web-based | Control |
| --- | --- | --- | --- |
| **Web-based sessions, n (%)** | *(n=36)* | *(n=33)* | *(n=31)* |
| **Ease of use** |  |  |  |
| Agree | 34 (94.4) | 31 (93.9) | 28 (90.3) |
| Neutral | 2 (5.6) | 2 (6.1) | 2 (6.5) |
| Disagree | 0 (0.0) | 0 (0.0) | 1(3.2) |
| **Understandability:** |  |  |  |
| Agree | 34 (94.4) | 30 (90.9) | 28 (90.3) |
| Neutral | 2 (5.6) | 3 (9.1) | 3 (9.7) |
| Disagree | 0 (0.0) | 0 (0.0) | 0 (0.0) |
| **Usefulness** |  |  |  |
| Agree | 32 (88.9) | 26 (78.8) | 26 (83.9) |
| Neutral | 4 (11.1) | 7 (21.2) | 4 (12.9) |
| Disagree | 0 (0.0) | 0 (0.0) | 1 (3.2) |
| **Enjoyability** |  |  |  |
| Agree | 30 (83.3) | 27 (81.8) | 24 (77.4) |
| Neutral | 6 (16.7) | 6 (18.2) | 7 (22.6) |
| Disagree | 0 (0.0) | 0 (0.0) | 0 (0.0) |
| **Acceptability of time** |  |  |  |
| Agree | 32 (88.9) | 30 (90.9) | 29 (93.5) |
| Neutral | 4 (11.1) | 3 (9.1) | 2 (6.5) |
| Disagree | 0 (0.0) | 0 (0.0) | 0 (0.0) |
| **Satisfaction** |  |  |  |
| Agree | 35 (97.2) | 32 (97.0) | 28 (90.3) |
| Neutral | 1 (2.8) | 1 (3.0) | 3 (9.7) |
| Disagree | 0 (0.0) | 0 (0.0) | 0 (0.0) |
| **E-workshop, n (%)** | *(n=25)* |  |  |
| **Clarity** |  |  |  |
| Agree | 24 (96.0) | N/A | N/A |
| Neutral | 1 (4.0) | N/A | N/A |
| Disagree | 0 (0.0) | N/A | N/A |
| **Understandability:** |  |  |  |
| Agree | 23 (92.0) | N/A | N/A |
| Neutral | 2 (8.0) | N/A | N/A |
| Disagree | 0 (0.0) | N/A | N/A |
| **Usefulness** |  |  |  |
| Agree | 22 (88.0) | N/A | N/A |
| Neutral | 3 (12.0) | N/A | N/A |
| Disagree | 0 (0.0) | N/A | N/A |
| **Enjoyability** |  |  |  |
| Agree | 23 (92.0) | N/A | N/A |
| Neutral | 2 (8.0) | N/A | N/A |
| Disagree | 0 (0.0) | N/A | N/A |
| **Acceptability of time** |  |  |  |
| Agree | 24 (96.0) | N/A | N/A |
| Neutral | 1 (4.0) | N/A | N/A |
| Disagree | 0 (0.0) | N/A | N/A |
| **Satisfaction** |  |  |  |
| Agree | 24 (96.0) | N/A | N/A |
| Neutral | 1 (4.0) | N/A | N/A |
| Disagree | 0 (0.0) | N/A | N/A |
