# Supplementary material for: Effectiveness of a Blended Intervention to Promote Physical Activity Among Office Employees: Randomized Controlled Trial
Source: J Med Internet Res. 2026 May 22;28:e80249. doi: 10.2196/80249 (PMC13197156; doi:10.2196/80249)
Supplement: Checklist 1 [file jmir-v28-e80249-s003.pdf]

CONSORT 2010 checklist of information to include when reporting a randomised trial*

Section/Topic	Item No	Checklist item	Reported on Page No
**Title and abstract**			
	1a	Identification as a randomised trial in the title	P1
	1b	Structured summary of trial design, methods, results, and conclusions (for specific guidance see CONSORT for abstracts)	P1-2
**Introduction**			
Background and objectives	2a	Scientific background and explanation of rationale	P3-5
	2b	Specific objectives or hypotheses	P4-5
**Methods**			
Trial design	3a	Description of trial design (such as parallel, factorial) including allocation ratio	P5
	3b	Important changes to methods after trial commencement (such as eligibility criteria), with reasons	P5
Participants	4a	Eligibility criteria for participants	P7
	4b	Settings and locations where the data were collected	P7
Interventions	5	The interventions for each group with sufficient details to allow replication, including how and when they were actually administered	P9-10
Outcomes	6a	Completely defined pre-specified primary and secondary outcome measures, including how and when they were assessed	P11

	6b	Any changes to trial outcomes after the trial commenced, with reasons	N/A
Sample size	7a	How sample size was determined	P8
	7b	When applicable, explanation of any interim analyses and stopping guidelines	N/A
Randomisation:			
Sequence generation	8a	Method used to generate the random allocation sequence	P8
	8b	Type of randomisation; details of any restriction (such as blocking and block size)	P8
Allocation concealment mechanism	9	Mechanism used to implement the random allocation sequence (such as sequentially numbered containers), describing any steps taken to conceal the sequence until interventions were assigned	P8
Implementation	10	Who generated the random allocation sequence, who enrolled participants, and who assigned participants to interventions	P8
Blinding	11a	If done, who was blinded after assignment to interventions (for example, participants, care providers, those assessing outcomes) and how	P8
	11b	If relevant, description of the similarity of interventions	N/A
Statistical methods	12a	Statistical methods used to compare groups for primary and secondary outcomes	P12
	12b	Methods for additional analyses, such as subgroup analyses and adjusted analyses	P12
**Results**			
Participant flow (a diagram is strongly recommended)	13a	For each group, the numbers of participants who were randomly assigned, received intended treatment, and were analysed for the primary outcome	P13 (with figure 1)
	13b	For each group, losses and exclusions after randomisation, together with reasons	P13
Recruitment	14a	Dates defining the periods of recruitment and follow-up	P13
	14b	Why the trial ended or was stopped	N/A

Baseline data	15	A table showing baseline demographic and clinical characteristics for each group	P14 (Table 1)
Numbers analysed	16	For each group, number of participants (denominator) included in each analysis and whether the analysis was by original assigned groups	P14
Outcomes and estimation	17a	For each primary and secondary outcome, results for each group, and the estimated effect size and its precision (such as 95% confidence interval)	P15-17 (Table 2 and 3)
	17b	For binary outcomes, presentation of both absolute and relative effect sizes is recommended	N/A
Ancillary analyses	18	Results of any other analyses performed, including subgroup analyses and adjusted analyses, distinguishing pre-specified from exploratory	P17
Harms	19	All important harms or unintended effects in each group (for specific guidance see CONSORT for harms)	P18
**Discussion**			
Limitations	20	Trial limitations, addressing sources of potential bias, imprecision, and, if relevant, multiplicity of analyses	P22
Generalisability	21	Generalisability (external validity, applicability) of the trial findings	P22
Interpretation	22	Interpretation consistent with results, balancing benefits and harms, and considering other relevant evidence	P18-21
**Other information**			
Registration	23	Registration number and name of trial registry	P2
Protocol	24	Where the full trial protocol can be accessed, if available	P24
Funding	25	Sources of funding and other support (such as supply of drugs), role of funders	P23
